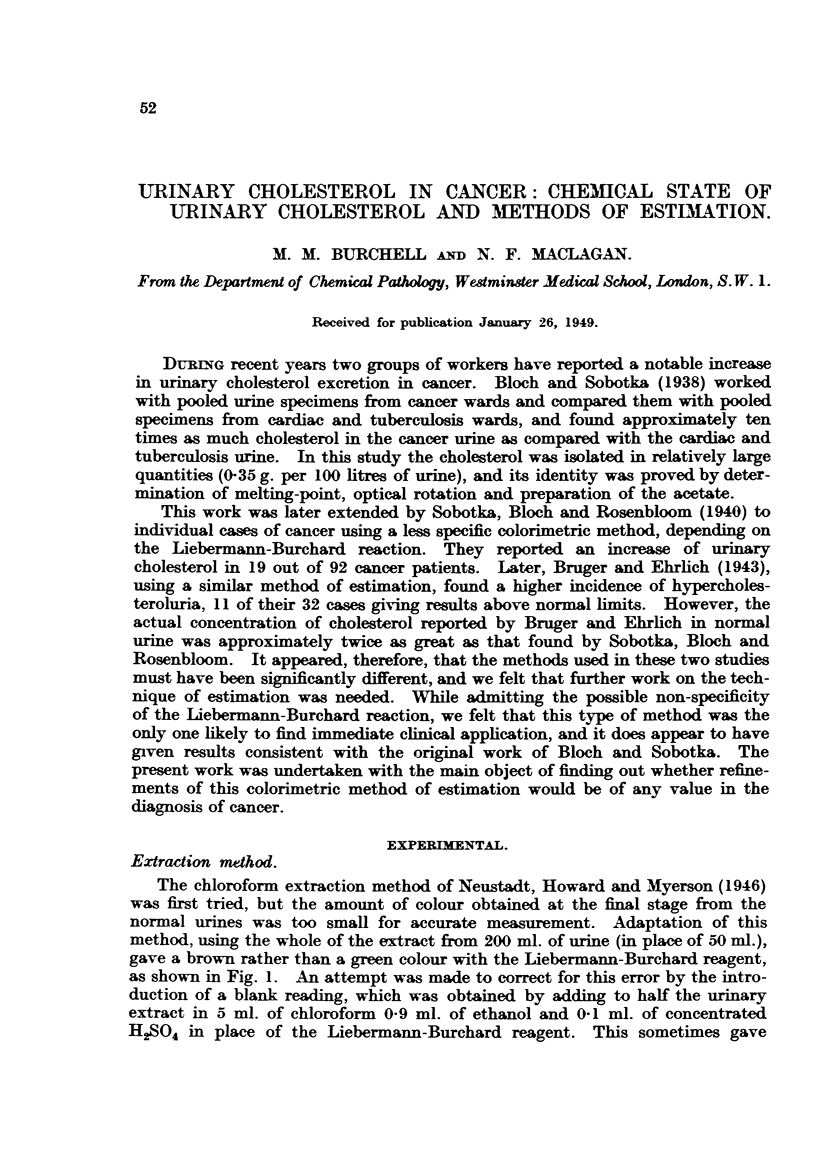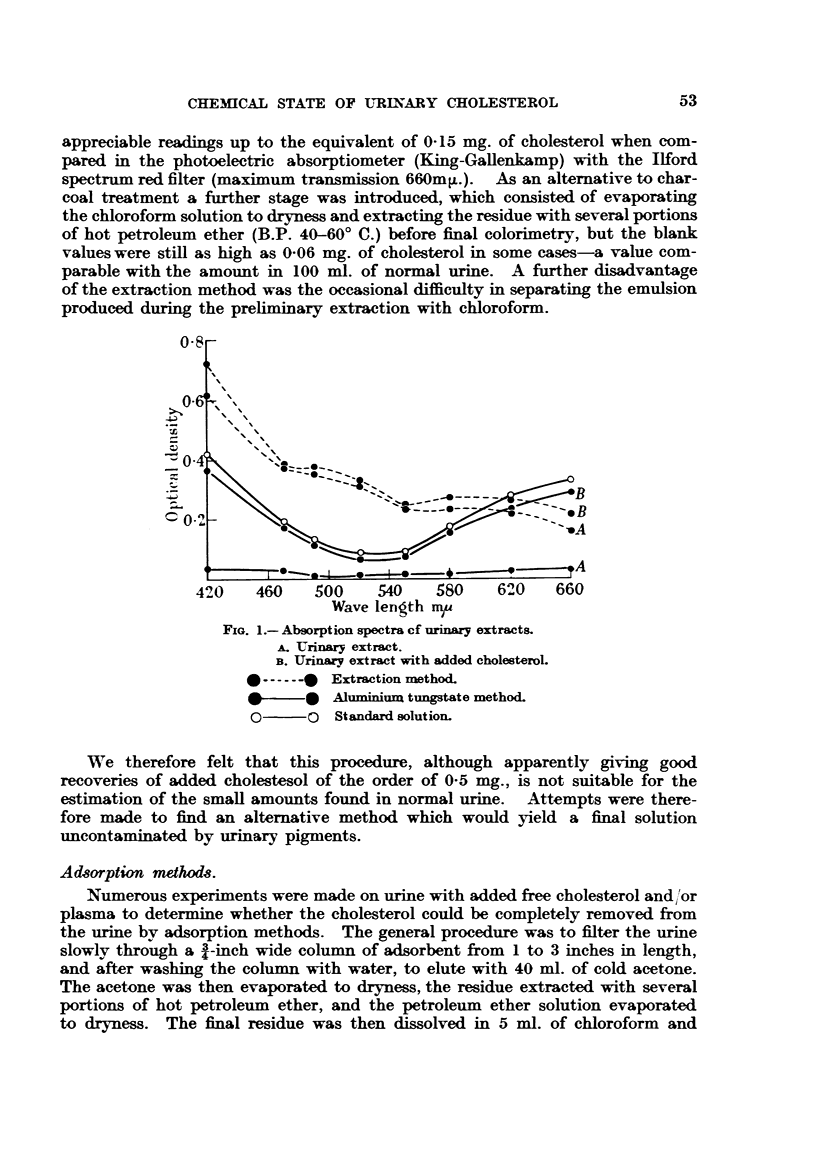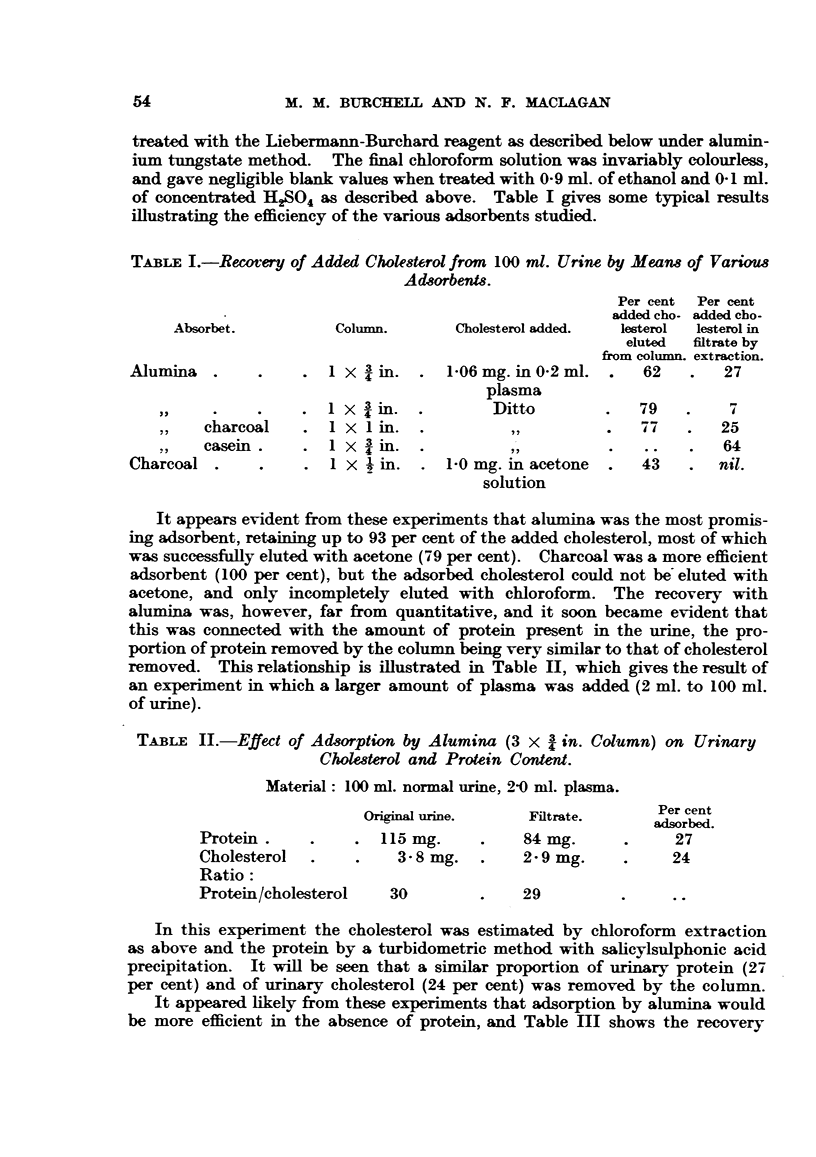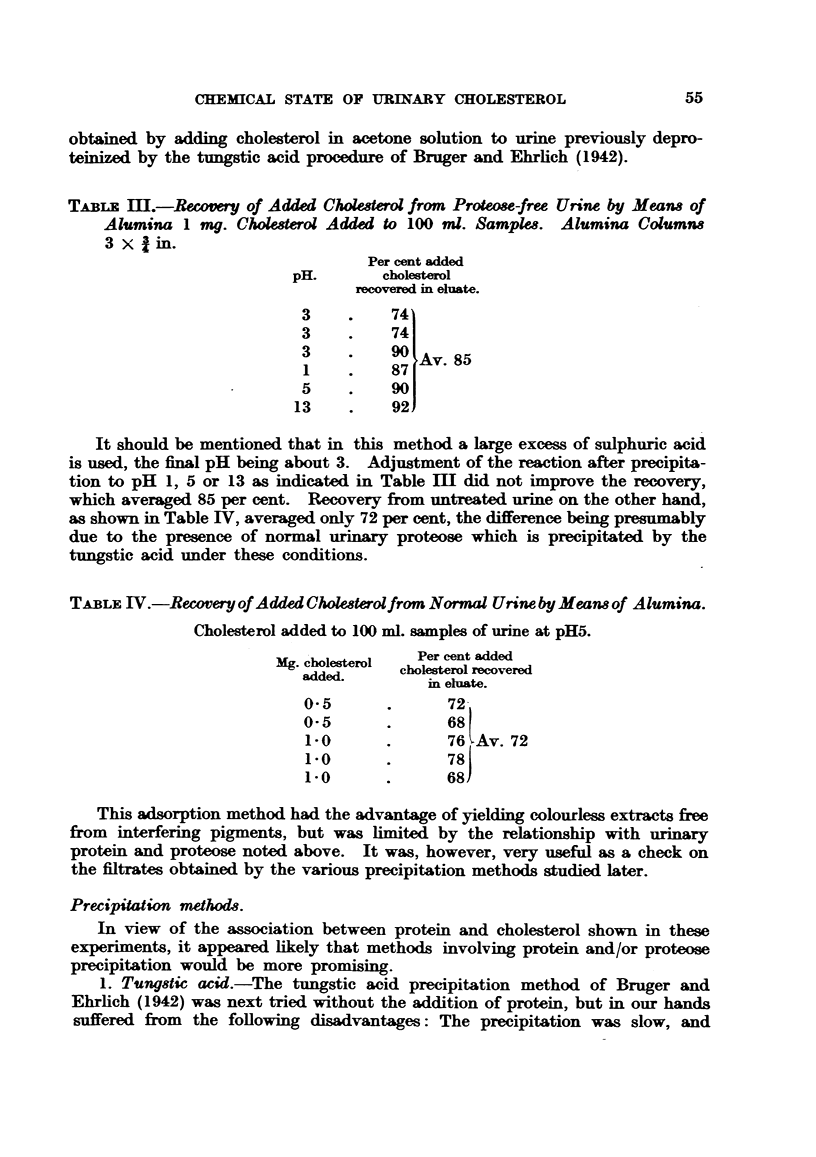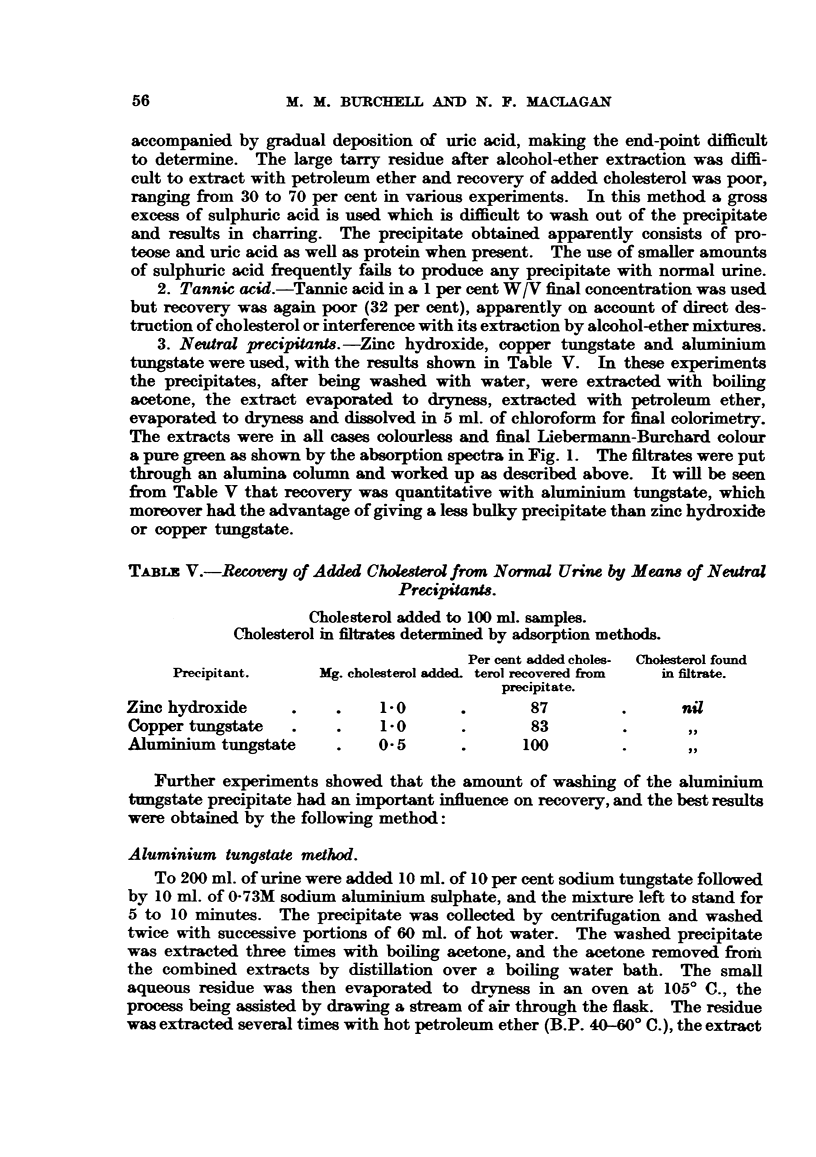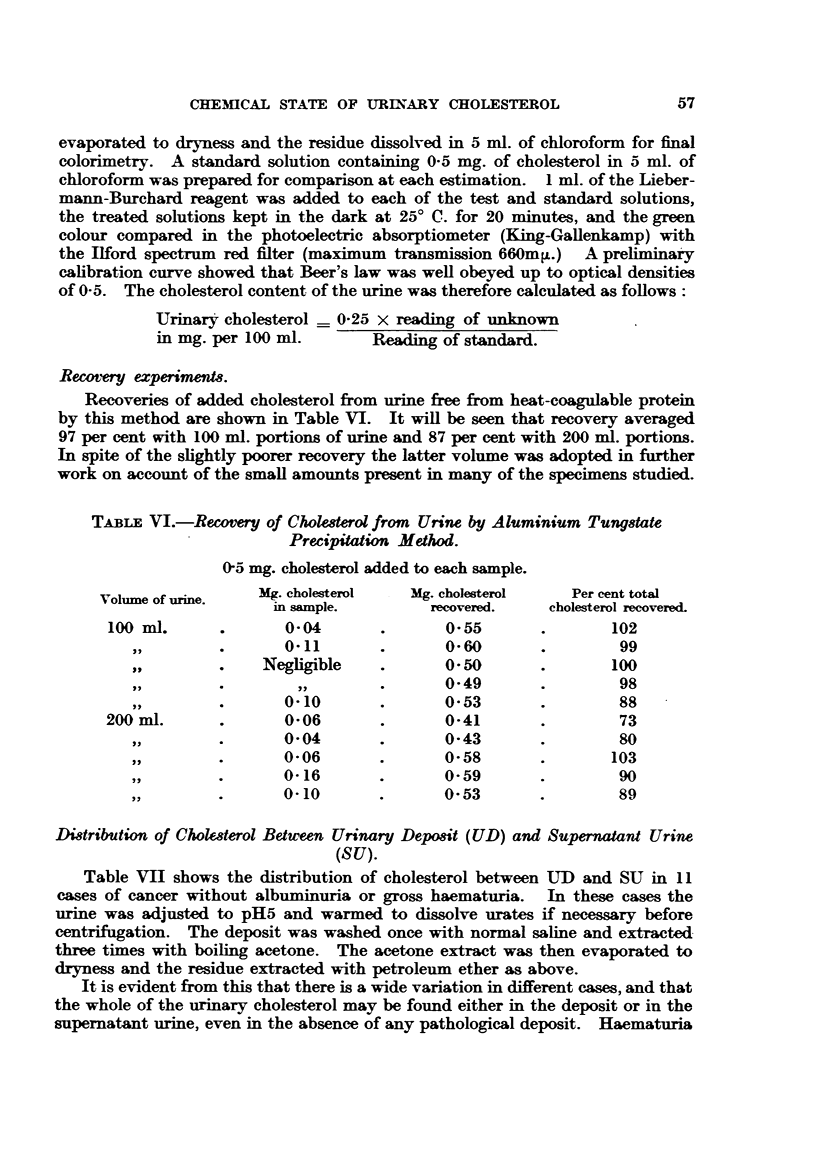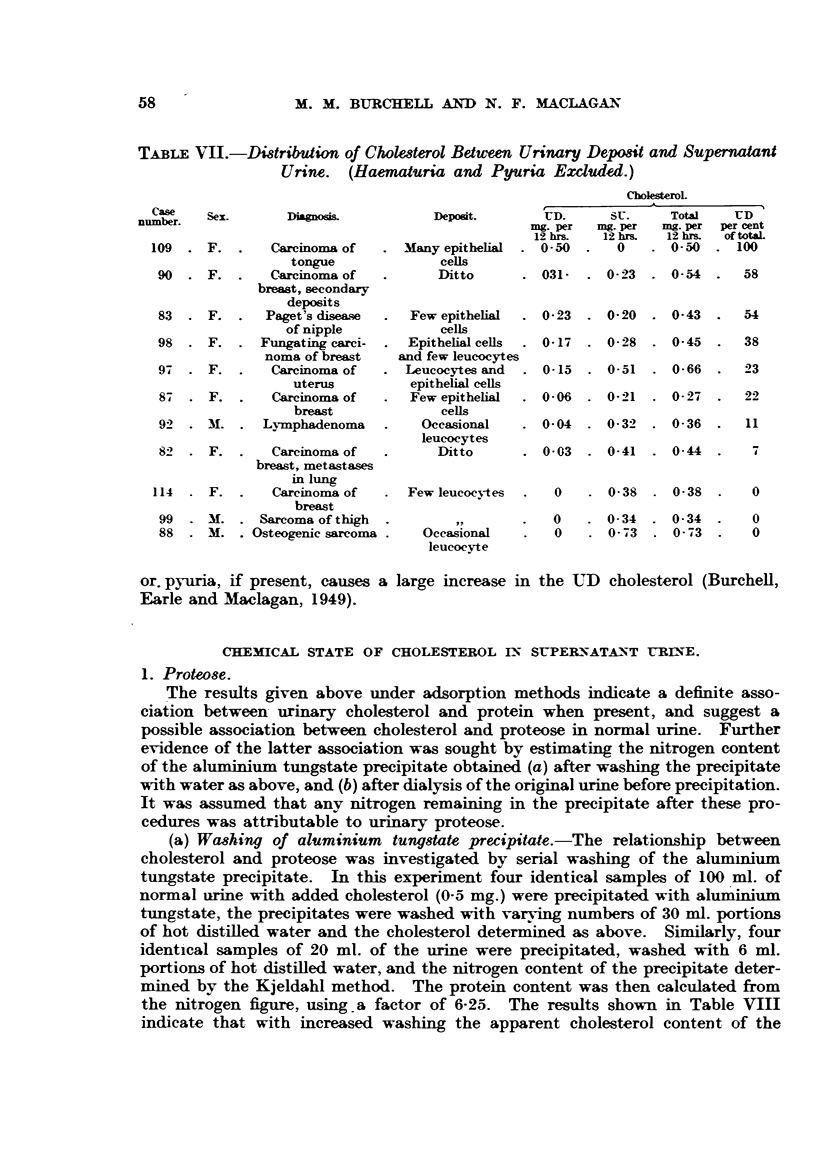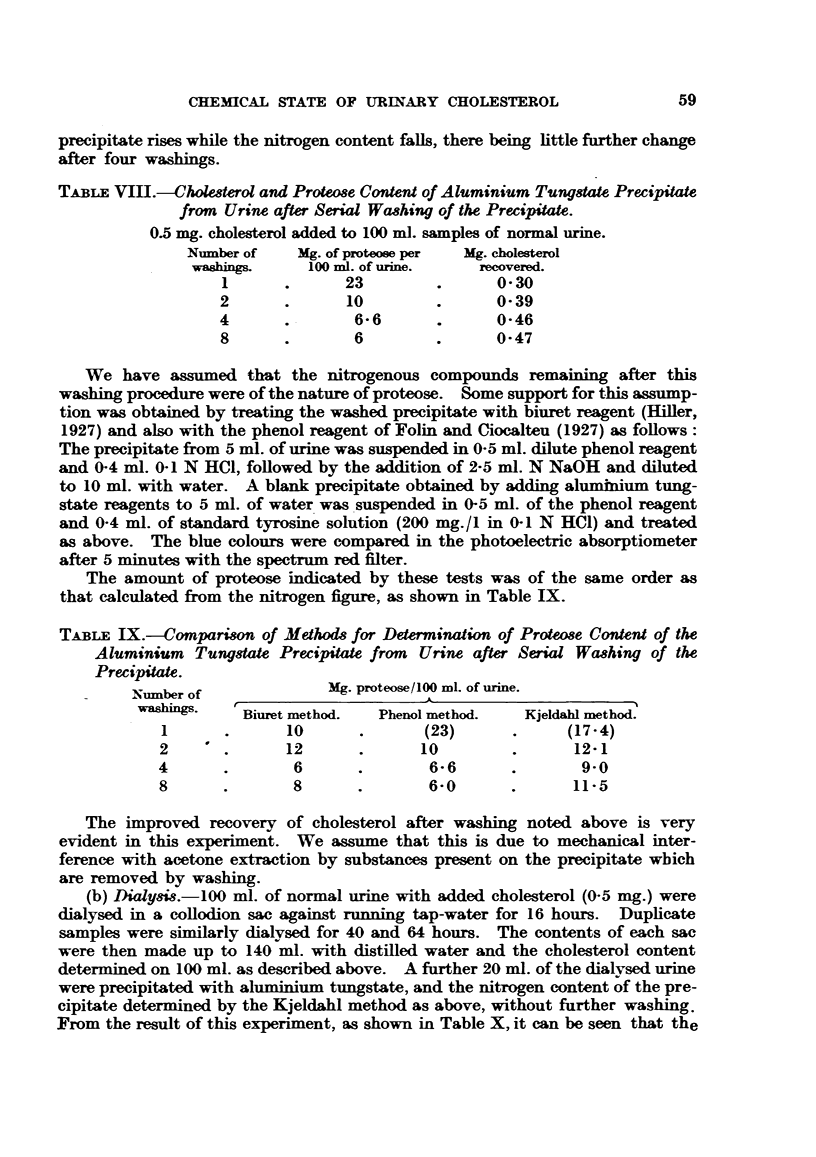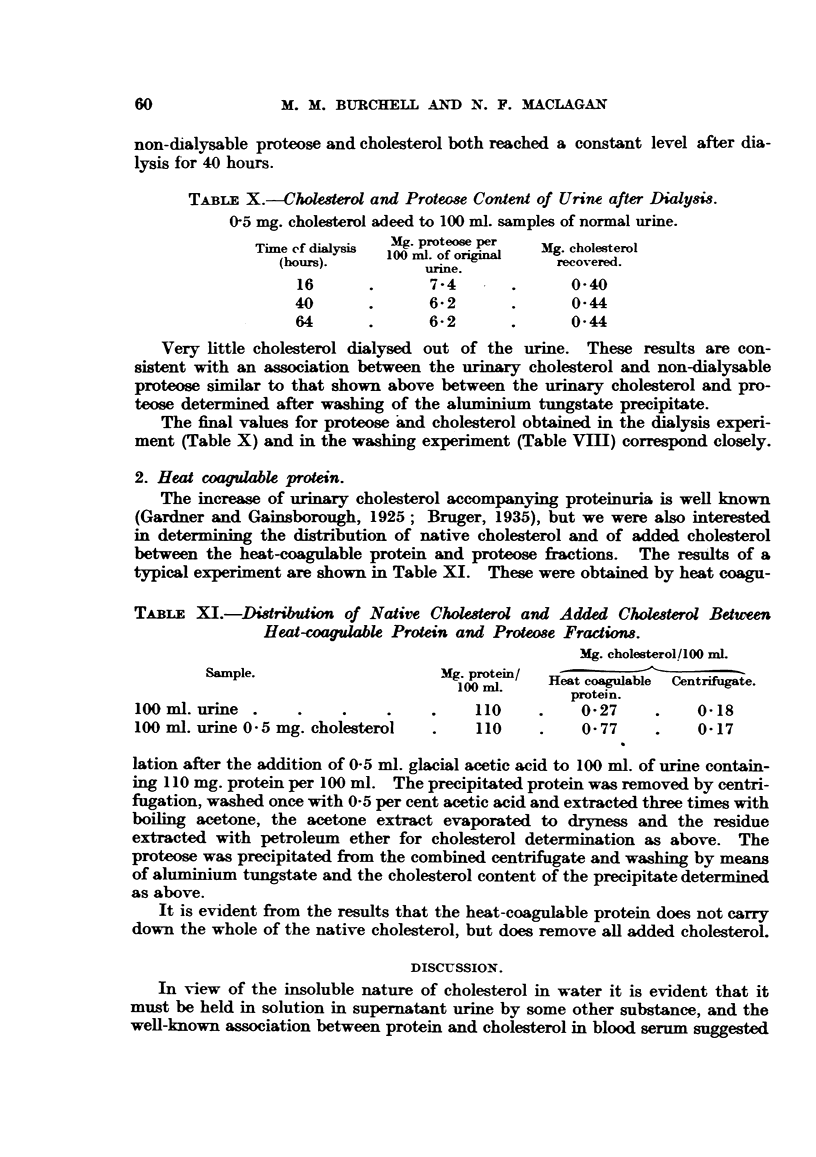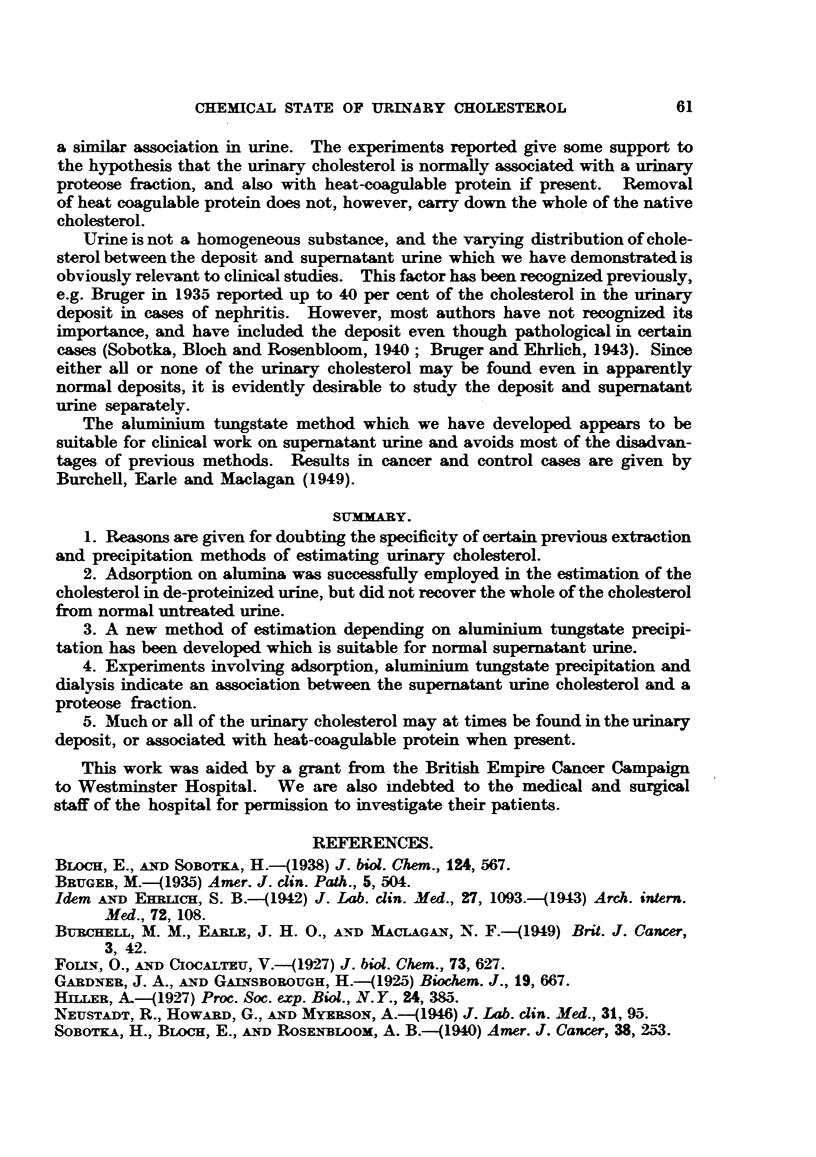# Urinary Cholesterol in Cancer: Chemical State of Urinary Cholesterol and Methods of Estimation

**Published:** 1949-03

**Authors:** M. M. Burchell, N. F. Maclagan


### URINARY CHOLESTEROL IN CANCER: CHEMICAL STATE OF URINARY CHOLESTEROL AND METHODS OF ESTIMATION.

M. M. BURCHELL AND N. F. MACLAGAN.

*From the Department of Chemical Pathology, Westminster Medical School, London, S.W. 1.*

Received for publication January 26, 1949.

DURING recent years two groups of workers have reported a notable increase in urinary cholesterol excretion in cancer. Bloch and Sobotka (1938) worked with pooled urine specimens from cancer wards and compared them with pooled specimens from cardiac and tuberculosis wards, and found approximately ten times as much cholesterol in the cancer urine as compared with the cardiac and tuberculosis urine. In this study the cholesterol was isolated in relatively large quantities (0·35 g. per 100 litres of urine), and its identity was proved by determination of melting-point, optical rotation and preparation of the acetate.

This work was later extended by Sobotka, Bloch and Rosenbloom (1940) to individual cases of cancer using a less specific colorimetric method, depending on the Liebermann-Burchard reaction. They reported an increase of urinary cholesterol in 19 out of 92 cancer patients. Later, Bruger and Ehrlich (1943), using a similar method of estimation, found a higher incidence of hypercholesteroluria, 11 of their 32 cases giving results above normal limits. However, the actual concentration of cholesterol reported by Bruger and Ehrlich in normal urine was approximately twice as great as that found by Sobotka, Bloch and Rosenbloom. It appeared, therefore, that the methods used in these two studies must have been significantly different, and we felt that further work on the technique of estimation was needed. While admitting the possible non-specificity of the Liebermann-Burchard reaction, we felt that this type of method was the only one likely to find immediate clinical application, and it does appear to have given results consistent with the original work of Bloch and Sobotka. The present work was undertaken with the main object of finding out whether refinements of this colorimetric method of estimation would be of any value in the diagnosis of cancer.

### EXPERIMENTAL.

### *Extraction method.*

The chloroform extraction method of Neustadt, Howard and Myerson (1946) was first tried, but the amount of colour obtained at the final stage from the normal urines was too small for accurate measurement. Adaptation of this method, using the whole of the extract from 200 ml. of urine (in place of 50 ml.), gave a brown rather than a green colour with the Liebermann-Burchard reagent, as shown in Fig. 1. An attempt was made to correct for this error by the introduction of a blank reading, which was obtained by adding to half the urinary extract in 5 ml. of chloroform 0·9 ml. of ethanol and 0·1 ml. of concentrated $H_2SO_4$ in place of the Liebermann-Burchard reagent. This sometimes gave appreciable readings up to the equivalent of 0·15 mg. of cholesterol when compared in the photoelectric absorptiometer (King-Gallenkamp) with the Ilford spectrum red filter (maximum transmission 660mμ.). As an alternative to charcoal treatment a further stage was introduced, which consisted of evaporating the chloroform solution to dryness and extracting the residue with several portions of hot petroleum ether (B.P. 40–60° C.) before final colorimetry, but the blank values were still as high as 0·06 mg. of cholesterol in some cases—a value comparable with the amount in 100 ml. of normal urine. A further disadvantage of the extraction method was the occasional difficulty in separating the emulsion produced during the preliminary extraction with chloroform.

FIG. 1.—Absorption spectra of urinary extracts.
A. Urinary extract.
B. Urinary extract with added cholesterol.
●- - - - - -● Extraction method.
●———● Aluminium tungstate method.
○———○ Standard solution.

We therefore felt that this procedure, although apparently giving good recoveries of added cholestesol of the order of 0·5 mg., is not suitable for the estimation of the small amounts found in normal urine. Attempts were therefore made to find an alternative method which would yield a final solution uncontaminated by urinary pigments.

*Adsorption methods.*

Numerous experiments were made on urine with added free cholesterol and/or plasma to determine whether the cholesterol could be completely removed from the urine by adsorption methods. The general procedure was to filter the urine slowly through a ¾-inch wide column of adsorbent from 1 to 3 inches in length, and after washing the column with water, to elute with 40 ml. of cold acetone. The acetone was then evaporated to dryness, the residue extracted with several portions of hot petroleum ether, and the petroleum ether solution evaporated to dryness. The final residue was then dissolved in 5 ml. of chloroform and treated with the Liebermann-Burchard reagent as described below under aluminium tungstate method. The final chloroform solution was invariably colourless, and gave negligible blank values when treated with 0·9 ml. of ethanol and 0·1 ml. of concentrated $H_2SO_4$ as described above. Table I gives some typical results illustrating the efficiency of the various adsorbents studied.

TABLE I.—*Recovery of Added Cholesterol from* 100 *ml. Urine by Means of Various Adsorbents.*

| Absorbet. | Column. | Cholesterol added. | Per cent added cholesterol eluted from column. | Per cent added cholesterol in filtrate by extraction. |
|---|---|---|---|---|
| Alumina | 1 × ¾ in. | 1·06 mg. in 0·2 ml. plasma | 62 | 27 |
| ,, | 1 × ¾ in. | Ditto | 79 | 7 |
| ,, charcoal | 1 × 1 in. | ,, | 77 | 25 |
| ,, casein | 1 × ¾ in. | ,, | .. | 64 |
| Charcoal | 1 × ½ in. | 1·0 mg. in acetone solution | 43 | *nil.* |

It appears evident from these experiments that alumina was the most promising adsorbent, retaining up to 93 per cent of the added cholesterol, most of which was successfully eluted with acetone (79 per cent). Charcoal was a more efficient adsorbent (100 per cent), but the adsorbed cholesterol could not be eluted with acetone, and only incompletely eluted with chloroform. The recovery with alumina was, however, far from quantitative, and it soon became evident that this was connected with the amount of protein present in the urine, the proportion of protein removed by the column being very similar to that of cholesterol removed. This relationship is illustrated in Table II, which gives the result of an experiment in which a larger amount of plasma was added (2 ml. to 100 ml. of urine).

TABLE II.—*Effect of Adsorption by Alumina* (3 × ¾ *in. Column*) *on Urinary Cholesterol and Protein Content.*

Material : 100 ml. normal urine, 2·0 ml. plasma.

| | Original urine. | Filtrate. | Per cent adsorbed. |
|---|---|---|---|
| Protein | 115 mg. | 84 mg. | 27 |
| Cholesterol | 3·8 mg. | 2·9 mg. | 24 |
| Ratio : | | | |
| Protein/cholesterol | 30 | 29 | .. |

In this experiment the cholesterol was estimated by chloroform extraction as above and the protein by a turbidometric method with salicylsulphonic acid precipitation. It will be seen that a similar proportion of urinary protein (27 per cent) and of urinary cholesterol (24 per cent) was removed by the column.

It appeared likely from these experiments that adsorption by alumina would be more efficient in the absence of protein, and Table III shows the recovery obtained by adding cholesterol in acetone solution to urine previously deproteinized by the tungstic acid procedure of Bruger and Ehrlich (1942).

TABLE III.—*Recovery of Added Cholesterol from Proteose-free Urine by Means of Alumina 1 mg. Cholesterol Added to 100 ml. Samples. Alumina Columns* 3 × ¾ in.

| pH. | Per cent added cholesterol recovered in eluate. | |
|---|---|---|
| 3 | 74 | Av. 85 |
| 3 | 74 | |
| 3 | 90 | |
| 1 | 87 | |
| 5 | 90 | |
| 13 | 92 | |

It should be mentioned that in this method a large excess of sulphuric acid is used, the final pH being about 3. Adjustment of the reaction after precipitation to pH 1, 5 or 13 as indicated in Table III did not improve the recovery, which averaged 85 per cent. Recovery from untreated urine on the other hand, as shown in Table IV, averaged only 72 per cent, the difference being presumably due to the presence of normal urinary proteose which is precipitated by the tungstic acid under these conditions.

TABLE IV.—*Recovery of Added Cholesterol from Normal Urine by Means of Alumina.*

Cholesterol added to 100 ml. samples of urine at pH5.

| Mg. cholesterol added. | Per cent added cholesterol recovered in eluate. | |
|---|---|---|
| 0·5 | 72 | Av. 72 |
| 0·5 | 68 | |
| 1·0 | 76 | |
| 1·0 | 78 | |
| 1·0 | 68 | |

This adsorption method had the advantage of yielding colourless extracts free from interfering pigments, but was limited by the relationship with urinary protein and proteose noted above. It was, however, very useful as a check on the filtrates obtained by the various precipitation methods studied later.

*Precipitation methods.*

In view of the association between protein and cholesterol shown in these experiments, it appeared likely that methods involving protein and/or proteose precipitation would be more promising.

1. *Tungstic acid.*—The tungstic acid precipitation method of Bruger and Ehrlich (1942) was next tried without the addition of protein, but in our hands suffered from the following disadvantages: The precipitation was slow, and accompanied by gradual deposition of uric acid, making the end-point difficult to determine. The large tarry residue after alcohol-ether extraction was difficult to extract with petroleum ether and recovery of added cholesterol was poor, ranging from 30 to 70 per cent in various experiments. In this method a gross excess of sulphuric acid is used which is difficult to wash out of the precipitate and results in charring. The precipitate obtained apparently consists of proteose and uric acid as well as protein when present. The use of smaller amounts of sulphuric acid frequently fails to produce any precipitate with normal urine.

2. *Tannic acid.*—Tannic acid in a 1 per cent W/V final concentration was used but recovery was again poor (32 per cent), apparently on account of direct destruction of cholesterol or interference with its extraction by alcohol-ether mixtures.

3. *Neutral precipitants.*—Zinc hydroxide, copper tungstate and aluminium tungstate were used, with the results shown in Table V. In these experiments the precipitates, after being washed with water, were extracted with boiling acetone, the extract evaporated to dryness, extracted with petroleum ether, evaporated to dryness and dissolved in 5 ml. of chloroform for final colorimetry. The extracts were in all cases colourless and final Liebermann-Burchard colour a pure green as shown by the absorption spectra in Fig. 1. The filtrates were put through an alumina column and worked up as described above. It will be seen from Table V that recovery was quantitative with aluminium tungstate, which moreover had the advantage of giving a less bulky precipitate than zinc hydroxide or copper tungstate.

TABLE V.—*Recovery of Added Cholesterol from Normal Urine by Means of Neutral Precipitants.*

Cholesterol added to 100 ml. samples.
Cholesterol in filtrates determined by adsorption methods.

| Precipitant. | Mg. cholesterol added. | Per cent added cholesterol recovered from precipitate. | Cholesterol found in filtrate. |
|---|---|---|---|
| Zinc hydroxide | 1·0 | 87 | *nil* |
| Copper tungstate | 1·0 | 83 | ,, |
| Aluminium tungstate | 0·5 | 100 | ,, |

Further experiments showed that the amount of washing of the aluminium tungstate precipitate had an important influence on recovery, and the best results were obtained by the following method:

*Aluminium tungstate method.*

To 200 ml. of urine were added 10 ml. of 10 per cent sodium tungstate followed by 10 ml. of 0·73M sodium aluminium sulphate, and the mixture left to stand for 5 to 10 minutes. The precipitate was collected by centrifugation and washed twice with successive portions of 60 ml. of hot water. The washed precipitate was extracted three times with boiling acetone, and the acetone removed from the combined extracts by distillation over a boiling water bath. The small aqueous residue was then evaporated to dryness in an oven at 105° C., the process being assisted by drawing a stream of air through the flask. The residue was extracted several times with hot petroleum ether (B.P. 40–60° C.), the extract evaporated to dryness and the residue dissolved in 5 ml. of chloroform for final colorimetry. A standard solution containing 0·5 mg. of cholesterol in 5 ml. of chloroform was prepared for comparison at each estimation. 1 ml. of the Liebermann-Burchard reagent was added to each of the test and standard solutions, the treated solutions kept in the dark at 25° C. for 20 minutes, and the green colour compared in the photoelectric absorptiometer (King-Gallenkamp) with the Ilford spectrum red filter (maximum transmission 660m$\mu$.) A preliminary calibration curve showed that Beer's law was well obeyed up to optical densities of 0·5. The cholesterol content of the urine was therefore calculated as follows :

$$\text{Urinary cholesterol in mg. per 100 ml.} = \frac{0{\cdot}25 \times \text{reading of unknown}}{\text{Reading of standard.}}$$

*Recovery experiments.*

Recoveries of added cholesterol from urine free from heat-coagulable protein by this method are shown in Table VI. It will be seen that recovery averaged 97 per cent with 100 ml. portions of urine and 87 per cent with 200 ml. portions. In spite of the slightly poorer recovery the latter volume was adopted in further work on account of the small amounts present in many of the specimens studied.

TABLE VI.—*Recovery of Cholesterol from Urine by Aluminium Tungstate Precipitation Method.*

0·5 mg. cholesterol added to each sample.

| Volume of urine. | Mg. cholesterol in sample. | Mg. cholesterol recovered. | Per cent total cholesterol recovered. |
|---|---|---|---|
| 100 ml. | 0·04 | 0·55 | 102 |
| ,, | 0·11 | 0·60 | 99 |
| ,, | Negligible | 0·50 | 100 |
| ,, | ,, | 0·49 | 98 |
| ,, | 0·10 | 0·53 | 88 |
| 200 ml. | 0·06 | 0·41 | 73 |
| ,, | 0·04 | 0·43 | 80 |
| ,, | 0·06 | 0·58 | 103 |
| ,, | 0·16 | 0·59 | 90 |
| ,, | 0·10 | 0·53 | 89 |

*Distribution of Cholesterol Between Urinary Deposit (UD) and Supernatant Urine (SU).*

Table VII shows the distribution of cholesterol between UD and SU in 11 cases of cancer without albuminuria or gross haematuria. In these cases the urine was adjusted to pH5 and warmed to dissolve urates if necessary before centrifugation. The deposit was washed once with normal saline and extracted three times with boiling acetone. The acetone extract was then evaporated to dryness and the residue extracted with petroleum ether as above.

It is evident from this that there is a wide variation in different cases, and that the whole of the urinary cholesterol may be found either in the deposit or in the supernatant urine, even in the absence of any pathological deposit. Haematuria

TABLE VII.—*Distribution of Cholesterol Between Urinary Deposit and Supernatant Urine. (Haematuria and Pyuria Excluded.)*

| Case number. | Sex. | Diagnosis. | Deposit. | Cholesterol. UD. mg. per 12 hrs. | SU. mg. per 12 hrs. | Total mg. per 12 hrs. | UD per cent of total. |
|---|---|---|---|---|---|---|---|
| 109 | F. | Carcinoma of tongue | Many epithelial cells | 0·50 | 0 | 0·50 | 100 |
| 90 | F. | Carcinoma of breast, secondary deposits | Ditto | 031· | 0·23 | 0·54 | 58 |
| 83 | F. | Paget's disease of nipple | Few epithelial cells | 0·23 | 0·20 | 0·43 | 54 |
| 98 | F. | Fungating carcinoma of breast | Epithelial cells and few leucocytes | 0·17 | 0·28 | 0·45 | 38 |
| 97 | F. | Carcinoma of uterus | Leucocytes and epithelial cells | 0·15 | 0·51 | 0·66 | 23 |
| 87 | F. | Carcinoma of breast | Few epithelial cells | 0·06 | 0·21 | 0·27 | 22 |
| 92 | M. | Lymphadenoma | Occasional leucocytes | 0·04 | 0·32 | 0·36 | 11 |
| 82 | F. | Carcinoma of breast, metastases in lung | Ditto | 0·03 | 0·41 | 0·44 | 7 |
| 114 | F. | Carcinoma of breast | Few leucocytes | 0 | 0·38 | 0·38 | 0 |
| 99 | M. | Sarcoma of thigh | " | 0 | 0·34 | 0·34 | 0 |
| 88 | M. | Osteogenic sarcoma | Occasional leucocyte | 0 | 0·73 | 0·73 | 0 |

or pyuria, if present, causes a large increase in the UD cholesterol (Burchell, Earle and Maclagan, 1949).

### CHEMICAL STATE OF CHOLESTEROL IN SUPERNATANT URINE.

1. *Proteose.*

The results given above under adsorption methods indicate a definite association between urinary cholesterol and protein when present, and suggest a possible association between cholesterol and proteose in normal urine. Further evidence of the latter association was sought by estimating the nitrogen content of the aluminium tungstate precipitate obtained (*a*) after washing the precipitate with water as above, and (*b*) after dialysis of the original urine before precipitation. It was assumed that any nitrogen remaining in the precipitate after these procedures was attributable to urinary proteose.

(a) *Washing of aluminium tungstate precipitate.*—The relationship between cholesterol and proteose was investigated by serial washing of the aluminium tungstate precipitate. In this experiment four identical samples of 100 ml. of normal urine with added cholesterol (0·5 mg.) were precipitated with aluminium tungstate, the precipitates were washed with varying numbers of 30 ml. portions of hot distilled water and the cholesterol determined as above. Similarly, four identical samples of 20 ml. of the urine were precipitated, washed with 6 ml. portions of hot distilled water, and the nitrogen content of the precipitate determined by the Kjeldahl method. The protein content was then calculated from the nitrogen figure, using a factor of 6·25. The results shown in Table VIII indicate that with increased washing the apparent cholesterol content of the precipitate rises while the nitrogen content falls, there being little further change after four washings.

TABLE VIII.—*Cholesterol and Proteose Content of Aluminium Tungstate Precipitate from Urine after Serial Washing of the Precipitate.*

0.5 mg. cholesterol added to 100 ml. samples of normal urine.

| Number of washings. | Mg. of proteose per 100 ml. of urine. | Mg. cholesterol recovered. |
|---|---|---|
| 1 | 23 | 0·30 |
| 2 | 10 | 0·39 |
| 4 | 6·6 | 0·46 |
| 8 | 6 | 0·47 |

We have assumed that the nitrogenous compounds remaining after this washing procedure were of the nature of proteose. Some support for this assumption was obtained by treating the washed precipitate with biuret reagent (Hiller, 1927) and also with the phenol reagent of Folin and Ciocalteu (1927) as follows: The precipitate from 5 ml. of urine was suspended in 0·5 ml. dilute phenol reagent and 0·4 ml. 0·1 N HCl, followed by the addition of 2·5 ml. N NaOH and diluted to 10 ml. with water. A blank precipitate obtained by adding aluminium tungstate reagents to 5 ml. of water was suspended in 0·5 ml. of the phenol reagent and 0·4 ml. of standard tyrosine solution (200 mg./l in 0·1 N HCl) and treated as above. The blue colours were compared in the photoelectric absorptiometer after 5 minutes with the spectrum red filter.

The amount of proteose indicated by these tests was of the same order as that calculated from the nitrogen figure, as shown in Table IX.

TABLE IX.—*Comparison of Methods for Determination of Proteose Content of the Aluminium Tungstate Precipitate from Urine after Serial Washing of the Precipitate.*

| Number of washings. | Mg. proteose/100 ml. of urine. | | |
|---|---|---|---|
| | Biuret method. | Phenol method. | Kjeldahl method. |
| 1 | 10 | (23) | (17·4) |
| 2 | 12 | 10 | 12·1 |
| 4 | 6 | 6·6 | 9·0 |
| 8 | 8 | 6·0 | 11·5 |

The improved recovery of cholesterol after washing noted above is very evident in this experiment. We assume that this is due to mechanical interference with acetone extraction by substances present on the precipitate which are removed by washing.

(b) *Dialysis.*—100 ml. of normal urine with added cholesterol (0·5 mg.) were dialysed in a collodion sac against running tap-water for 16 hours. Duplicate samples were similarly dialysed for 40 and 64 hours. The contents of each sac were then made up to 140 ml. with distilled water and the cholesterol content determined on 100 ml. as described above. A further 20 ml. of the dialysed urine were precipitated with aluminium tungstate, and the nitrogen content of the precipitate determined by the Kjeldahl method as above, without further washing. From the result of this experiment, as shown in Table X, it can be seen that the non-dialysable proteose and cholesterol both reached a constant level after dialysis for 40 hours.

TABLE X.—*Cholesterol and Proteose Content of Urine after Dialysis.*

0·5 mg. cholesterol adeed to 100 ml. samples of normal urine.

| Time cf dialysis (hours). | Mg. proteose per 100 ml. of original urine. | Mg. cholesterol recovered. |
|---|---|---|
| 16 | 7·4 | 0·40 |
| 40 | 6·2 | 0·44 |
| 64 | 6·2 | 0·44 |

Very little cholesterol dialysed out of the urine. These results are consistent with an association between the urinary cholesterol and non-dialysable proteose similar to that shown above between the urinary cholesterol and proteose determined after washing of the aluminium tungstate precipitate.

The final values for proteose and cholesterol obtained in the dialysis experiment (Table X) and in the washing experiment (Table VIII) correspond closely.

2. *Heat coagulable protein.*

The increase of urinary cholesterol accompanying proteinuria is well known (Gardner and Gainsborough, 1925; Bruger, 1935), but we were also interested in determining the distribution of native cholesterol and of added cholesterol between the heat-coagulable protein and proteose fractions. The results of a typical experiment are shown in Table XI. These were obtained by heat coagu-

TABLE XI.—*Distribution of Native Cholesterol and Added Cholesterol Between Heat-coagulable Protein and Proteose Fractions.*

| Sample. | Mg. protein/ 100 ml. | Mg. cholesterol/100 ml. | |
|---|---|---|---|
| | | Heat coagulable protein. | Centrifugate. |
| 100 ml. urine | 110 | 0·27 | 0·18 |
| 100 ml. urine 0·5 mg. cholesterol | 110 | 0·77 | 0·17 |

lation after the addition of 0·5 ml. glacial acetic acid to 100 ml. of urine containing 110 mg. protein per 100 ml. The precipitated protein was removed by centrifugation, washed once with 0·5 per cent acetic acid and extracted three times with boiling acetone, the acetone extract evaporated to dryness and the residue extracted with petroleum ether for cholesterol determination as above. The proteose was precipitated from the combined centrifugate and washing by means of aluminium tungstate and the cholesterol content of the precipitate determined as above.

It is evident from the results that the heat-coagulable protein does not carry down the whole of the native cholesterol, but does remove all added cholesterol.

### DISCUSSION.

In view of the insoluble nature of cholesterol in water it is evident that it must be held in solution in supernatant urine by some other substance, and the well-known association between protein and cholesterol in blood serum suggested a similar association in urine. The experiments reported give some support to the hypothesis that the urinary cholesterol is normally associated with a urinary proteose fraction, and also with heat-coagulable protein if present. Removal of heat coagulable protein does not, however, carry down the whole of the native cholesterol.

Urine is not a homogeneous substance, and the varying distribution of cholesterol between the deposit and supernatant urine which we have demonstrated is obviously relevant to clinical studies. This factor has been recognized previously, e.g. Bruger in 1935 reported up to 40 per cent of the cholesterol in the urinary deposit in cases of nephritis. However, most authors have not recognized its importance, and have included the deposit even though pathological in certain cases (Sobotka, Bloch and Rosenbloom, 1940 ; Bruger and Ehrlich, 1943). Since either all or none of the urinary cholesterol may be found even in apparently normal deposits, it is evidently desirable to study the deposit and supernatant urine separately.

The aluminium tungstate method which we have developed appears to be suitable for clinical work on supernatant urine and avoids most of the disadvantages of previous methods. Results in cancer and control cases are given by Burchell, Earle and Maclagan (1949).

### SUMMARY.

1. Reasons are given for doubting the specificity of certain previous extraction and precipitation methods of estimating urinary cholesterol.
2. Adsorption on alumina was successfully employed in the estimation of the cholesterol in de-proteinized urine, but did not recover the whole of the cholesterol from normal untreated urine.
3. A new method of estimation depending on aluminium tungstate precipitation has been developed which is suitable for normal supernatant urine.
4. Experiments involving adsorption, aluminium tungstate precipitation and dialysis indicate an association between the supernatant urine cholesterol and a proteose fraction.
5. Much or all of the urinary cholesterol may at times be found in the urinary deposit, or associated with heat-coagulable protein when present.

This work was aided by a grant from the British Empire Cancer Campaign to Westminster Hospital. We are also indebted to the medical and surgical staff of the hospital for permission to investigate their patients.

### REFERENCES.

BLOCH, E., AND SOBOTKA, H.—(1938) *J. biol. Chem.*, **124**, 567.

BRUGER, M.—(1935) *Amer. J. clin. Path.*, **5**, 504.

*Idem* AND EHRLICH, S. B.—(1942) *J. Lab. clin. Med.*, **27**, 1093.—(1943) *Arch. intern. Med.*, **72**, 108.

BURCHELL, M. M., EARLE, J. H. O., AND MACLAGAN, N. F.—(1949) *Brit. J. Cancer*, **3**, 42.

FOLIN, O., AND CIOCALTEU, V.—(1927) *J. biol. Chem.*, **73**, 627.

GARDNER, J. A., AND GAINSBOROUGH, H.—(1925) *Biochem. J.*, **19**, 667.

HILLER, A.—(1927) *Proc. Soc. exp. Biol., N.Y.*, **24**, 385.

NEUSTADT, R., HOWARD, G., AND MYERSON, A.—(1946) *J. Lab. clin. Med.*, **31**, 95.

SOBOTKA, H., BLOCH, E., AND ROSENBLOOM, A. B.—(1940) *Amer. J. Cancer*, **38**, 253.